# Prognostic Impact of Baseline Neutrophil-to-Eosinophil Ratio in Patients With Metastatic Renal Cell Carcinoma Treated With Nivolumab Therapy in Second or Later Lines

**DOI:** 10.7759/cureus.22224

**Published:** 2022-02-15

**Authors:** Lucia Gil, Fátima R Alves, Diana Silva, Isabel Fernandes, Mário Fontes-Sousa, Marta Alves, Ana Papoila, Ricardo Da Luz

**Affiliations:** 1 Medical Oncology, Centro Hospitalar Universitário Lisboa Central, Lisboa, PRT; 2 Medical Oncology, Centro Hospitalar de Lisboa Ocidental, Lisboa, PRT; 3 Medical Oncology, Hospital Beatriz Ângelo, Loures, PRT; 4 Medical Oncology, Centro Hospitalar Barreiro Montijo, Barreiro, PRT; 5 Medical Oncology, Hospital CUF Tejo, Lisboa, PRT; 6 Epidemiology and Statistics Unit, Research Centre, Centro Hospitalar Universitário Lisboa Central, Lisboa, PRT

**Keywords:** progression-free survival, overall survival, prognostic, biomarker, neutrophil-to-eosinophil ratio, nivolumab, metastatic renal cell carcinoma

## Abstract

Background

Inflammation is a crucial component in carcinogenesis. The neutrophil-to-eosinophil ratio (NER) has been studied as a biomarker of prognosis and predictive of response in metastatic renal cell carcinoma (mRCC). In the present study, we evaluated the relevance of baseline NER on the progression-free survival (PFS) and overall survival (OS) outcomes in real-world patients with mRCC treated with nivolumab in second or subsequent lines. We also assessed the association of baseline NER with objective response, as well as with toxicity and histology.

Methods

In this multicenter retrospective analysis of patients with mRCC treated with nivolumab, the last systemic absolute neutrophil and eosinophil count before treatment with nivolumab was used to calculate the NER. An additive Cox proportional hazards model was used to identify the cut-off point for NER considering PFS and the patients were allocated into low and high NER groups. Median OS and median PFS were estimated using the Kaplan-Meier estimator, and survival curves of groups were compared using the log-rank test. Univariable and multivariable Cox regression models were used to study OS and PFS and Fisher’s exact test was performed to evaluate the association of NER with the response, toxicity, and histology.

Results

The 49 analyzed patients had a median follow-up of nine months. The NER cut-off was established at 48, locating 29 patients in the low NER group (NER < 48) and 20 in the high NER group (NER ≥ 48). Median PFS and median OS were significantly shorter in patients with high NER versus low NER (3 vs. 30 months (p < 0.001) and 6 vs. 24 months (p = 0.002), respectively). Multivariable analyses showed that NER (HR 3.92 (95% CI: 1.66-9.23), p = 0.002) was an independent factor for PFS and that NER (HR 3.85 (95% CI: 1.33-11.17), p = 0.013) and progressive disease (HR 5.62 (95% CI: 1.88-16.83), p = 0.002) were independent factors for OS. NER was significantly associated with objective response rate (ORR) (NER ≥ 48-12.5% vs. NER < 48-87.5%, p = 0.003), immune-related adverse events (irAEs) (NER ≥ 48-10.0% vs. NER < 48-42.9%, p = 0.014), and tumor’s histology as patients of high NER group had more non-clear cell carcinoma than low NER group (35.0% vs. 7.4%, p = 0.017).

Conclusion

Our real-world data analysis of NER in patients with mRCC confirmed the prognostic value of this biomarker, supporting clinical utility in predicting survival. Results also suggested an association between lower NER and better ORR, and that irAEs occur more frequently in patients with a lower NER. However, further large-scale prospective studies are needed to confirm these findings and to validate this biomarker.

## Introduction

Renal cell carcinoma (RCC) accounts for 3%-5% of all malignancies, representing the seventh most common cancer in men, and the 10th most common cancer in women [1]. In 2020, around 431,000 new cases have been diagnosed around the world [2]. There is an increasing incidence, especially in developed countries. Given the usual absence of symptoms, RCC is diagnosed at a metastatic stage in 30-50% of patients, with a poor prognosis due to estimated five-year survival of 10% [2].

RCC is a chemo-resistant disease and was also considered radiotherapy-resistant before the emergence of stereotactic radiation therapy [3,4]. Treatment for a long time was exclusively surgical. It was only later on that immunotherapy or targeted therapies showed survival benefits, the latter with a good toxicity profile [3,4]. A variety of targeted therapies, such as vascular endothelial growth factor (VEGF) monoclonal antibodies, tyrosine kinase inhibitors (TKIs), and mammalian target of rapamycin pathway inhibitors (mTORis), have been approved as systemic therapy in metastatic renal cell carcinoma (mRCC) [5]. Currently, immunotherapy has gained an important place in the treatment of this disease.

Immune checkpoint inhibitors (ICIs) have changed the treatment strategy for mRCC [6]. Nivolumab is an anti-programmed cell death protein 1 (PD-1) antibody, which is the standard second-line treatment in mRCC, after previous VEGF receptor TKI (VEGFR-TKI), and was approved based on the CheckMate 025 study, a phase III randomized clinical trial, which demonstrated improved overall survival (OS) compared to everolimus, regardless of programmed death-ligand 1 (PD-L1) expression with good toxicity profile [7]. The survival benefit was independent of PD-L1 expression [8]. The CheckMate 214 trial has demonstrated that the combination of nivolumab plus ipilimumab as first-line treatment in mRCC is associated with better OS and objective response rate (ORR), compared to sunitinib, among patients with intermediate and poor prognosis, according to the International Metastatic Renal Cell Carcinoma Database Consortium (IMDC) risk category [9]. Other studies have evaluated combinations of immunotherapy and TKIs as the first-line of treatment in mRCC, namely, the combination of nivolumab plus cabozantinib in CheckMate 9ER or pembrolizumab plus axitinib in KEYNOTE-426 [10,11]. Both clinical trials highlighted improvements in progression-free survival (PFS) and OS for the combinations [10,11]. These associations have now earned a place in standard first-line treatment in mRCC [12].

Consistent and long-lasting responses are seen in a group of patients treated with nivolumab. However, for another group of patients, the benefit of this therapy is limited (20-35%) [13]. The IMDC risk categories were developed using data from patients who received targeted therapy [14]. Thus, novel prognostic biomarkers or models are required in the ICIs era and it is essential to define biomarkers predicting the clinical outcome of ICIs treatment that can be routinely used in clinical practice [15].

Inflammation impacts each step of carcinogenesis, including tumor initiation, promotion, and metastatic progression, and ongoing studies are being carried out to find and select predictive markers, such as gene expression signatures, tumor mutational burden, or tumor-infiltrating lymphocytes [16-19]. However, serum markers are more practical and accessible biomarkers in routine clinical practice; many biomarkers of inflammation, including C-reactive protein (CRP) levels, neutrophil-to-lymphocyte ratio (NLR), eosinophil, and platelet count, have been investigated as prognostic factors [5,16,20].

A retrospective study demonstrated that an increase in eosinophils and relative eosinophil change at six weeks of nivolumab was associated with a good response to immunotherapy [5]. The baseline neutrophil-to-eosinophil ratio (NER) has been reported to be associated with outcomes of immuno-oncology based on combination treatment in mRCC, and a post hoc analysis of a phase III randomized control study, JAVELIN Renal 101, showed that a lower NER was associated with a better ORR and PFS with the combination of avelumab (anti-PD-L1) and axitinib in mRCC [21]. The fact that the early eosinophilia may lead to a better response to immunotherapy can be explained by the results presented by Cheng et al., who showed that the increase in eosinophils was involved with the recruitment of cluster of differentiation 8 (CD8) + T lymphocytes to the tumor microenvironment (TME) and that eosinophils contribute to the cytotoxic antitumor response [22].

The aim of the present study was to evaluate the relevance of baseline NER on the PFS and OS outcomes in real-world patients with mRCC treated with nivolumab in second or subsequent lines. Secondary purposes were to study the association of baseline NER to objective response through imaging exams, according to the Response Evaluation Criteria in Solid Tumors (RECIST) 1.1, as well as with toxicities and histology.

## Materials and methods

Patients

This multicenter retrospective cohort study considered patients with mRCC who were treated with nivolumab as second or subsequent lines at the medical oncology units of Centro Hospitalar Universitário Lisboa Central, Centro Hospitalar de Lisboa Ocidental, Centro Hospitalar Barreiro Montijo, and Hospital Beatriz Ângelo in Portugal, between June 2017 and April 2021. Patients who received immunotherapy as the first line and with other concomitant primary neoplasia(s) were excluded. Baseline patient and tumor characteristics, evidence of metastasis at diagnosis, history of surgical resection of metastases or primary tumor, first-line treatment, and response were all retrospectively collected from the hospital’s electronic database and medical records. The last follow-up data were collected as of 28 November 2021. The treatment response was evaluated by computed tomography (CT) and/or bone scintigraphy at least once every 12 weeks and classified according to RECIST 1.1. The ORR was defined as the percentage of patients with confirmed complete or partial responses, and clinical benefit was defined as the percentage of patients with confirmed complete or partial responses or stable disease among all treated patients. The study protocol was approved by the Research Ethics Committee of all the institutions and applied in conformity with the Declaration of Helsinki. A formal informed consent waiver was accepted due to the retrospective observational nature of this study.

Blood sample analysis

Values were obtained from clinical practice blood samples analysis. Peripheral blood parameters were collected during the week before the first administration of nivolumab as second or subsequent lines. The baseline NER was calculated from the counts obtained using that sample, dividing the absolute neutrophil count by the absolute eosinophil count.

Statistics

Descriptive statistics were performed with categorical variables being described as frequencies (percentages), and the remaining variables by the median and range (minimum and maximum). An additive Cox proportional hazards model was used to identify the cut-off point for NER considering PFS. Based on this cut-off value, patients were allocated into two groups. The OS and PFS times were calculated from the start of the treatment with nivolumab to the death from any cause, or to the progression of disease or death, respectively. Median OS (mOS) and median PFS (mPFS) were estimated using the Kaplan-Meier estimator, and survival curves of groups were compared using the log-rank test. Univariable and multivariable Cox regression models were used to study OS and PFS. For the multivariable models, all variables that in the univariable analysis attained a p-value ≤ 0.25 were selected. Adjusted hazard ratios (HRs) were estimated with corresponding 95% confidence intervals (95% CI). The proportional hazards assumption of Cox regression was tested with a formal significance test based on standardized Schoenfeld residuals. Fisher’s exact test was performed to evaluate the association of the baseline binary NER with the response, toxicity, and histology. A level of significance α = 0.05 was considered. Data analysis was performed using SPSS version 25.0 (IBM Corp., Armonk, NY) and R (R Foundation for Statistical Computing, Vienna, Austria).

## Results

Patients characteristics

The study included 49 patients with mRCC treated between June 2017 and April 2021 in the four institutions included in this study. Median follow-up was nine months (range: 1-57 months). Table 1 details patient demographics and baseline characteristics. Briefly, the median age at diagnosis of metastatic disease in this cohort was 61 years (range: 28-85 years) and 42 patients (85.7%) were male. Thirty-eight patients (77.6%) were diagnosed with a clear cell histotype. Based on the IMDC risk categories [23], nine (18.4%), 34 (69.4%), and six (12.2%) patients were categorized as favorable, intermediate, and poor risk, respectively. Nivolumab was administered in the third or subsequent lines in eight patients (16.3%), and all previously targeted therapies in first-line and most (n = 6) in second-line therapies were TKIs. The median NER was 33 with a range of 3-803. The NER cut-off in our cohort was 48 for the time to disease progression in patients with nivolumab therapy (sensitivity, 53.8%; specificity, 71.4%; Supplemental Figure). The NER was 48 or higher in 20 patients (40.8%, high-risk group) and it was less than 48 in 29 patients (59.2%, low-risk group).

**Table 1:** Patients' demographics and baseline characteristics. PS ECOG - Eastern Cooperative Oncology Group Performance Status Scale; IMDC - International Metastatic Renal Cell Carcinoma Database Consortium; TKIs - tyrosine kinase inhibitors; mTORis - mammalian target of rapamycin inhibitors; NER - neutrophil-to-eosinophil ratio; irAE - immune-related adverse events.

Characteristic	All (n = 49) (100%)
Age (years)	
Median	61
Range	28-85
Sex, n (%)	
Men	42 (85.7)
Women	7 (14.3)
PS ECOG, n (%)	
0	31 (63.3)
1	17 (34.7)
2	1 (2.0)
Previous nephrectomy, n (%)	
Yes	39 (79.6)
No	10 (20.4)
Histopathology, n (%)	
Clear cell carcinoma	38 (77.6)
Non-clear cell carcinoma	9 (18.4)
Missing	2 (4.1)
Fuhrman's grade, n (%)	
1, 2	20 (40.8)
3	10 (20.4)
4	5 (10.2)
Missing	14 (28.6)
IMDC risk, n (%)	
Favorable	9 (18.4)
Intermediate	34 (69.4)
Poor	6 (12.2)
Number of previously targeted therapies, n (%)	
1	42 (85.7)
≥2	7 (14.3)
Previously targeted therapies, n (%)	
TKIs	49 (100)
Sunitinib	29 (59.2)
Pazopanib	20 (40.8)
Axitinib	7 (14.3)
mTORis	2 (4.1)
Everolimus	2 (4.1)
Number of metastatic organs, n (%)	
1	25 (51.0)
≥2	24 (49.0)
NER	
<48	29 (59.2)
≥48	20 (40.8)
Occurrence of irAE, n (%)	
Yes	14 (28.6)
No	35 (71.4)

Treatment response and survival outcomes

**Figure 1:**
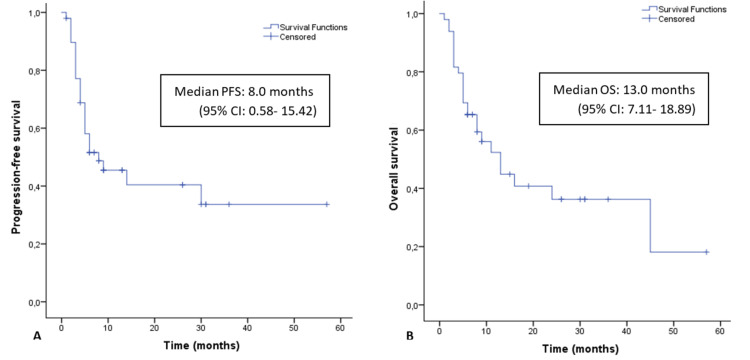
(A) Progression-free survival and (B) overall survival among mRCC patients treated with nivolumab in second or later lines estimates by Kaplan-Meier. PFS - progression-free survival; OS - overall survival; CI - confidence interval.

The response assessment to nivolumab was available in 48 of 49 patients because one patient died before the response assessment from a cause unrelated to the mRCC. Of these, one patient achieved a complete response, and 15 achieved a partial response. The ORR was 32.7% and the clinical benefit was 42.9% (Table 2). Twenty-seven patients (55.1%) experienced disease progression during treatment with nivolumab. The median PFS of nivolumab was eight months (95% CI: 0.58-15.42 months), and the median OS was 13.0 months (95% CI: 7.11-18.89 months) during the median nine months of follow up in this study (Figures 1A, 1B).

**Table 2:** Treatment response in mRCC patients treated with nivolumab in second or later lines. CR - complete response; PR - partial response; SD - stable disease; PD - progressive disease.

Treatment response	n (%)
Objective response	16 (32.7)
CR	1 (2.0)
PR	15 (30.6)
SD	5 (10.2)
PD	27 (55.1)
Unable to determine	1 (2.0)

Survival outcomes according to baseline NER and risk factors for survival

**Figure 2:**
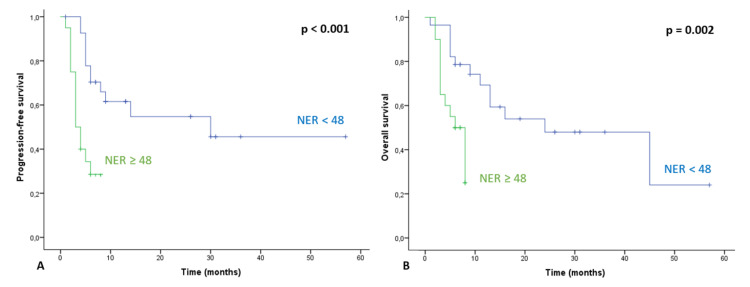
(A) Progression-free survival according to baseline NER. Higher NER (≥48) was significantly associated with shorter median PFS (3.0 vs. 30.0 months, p < 0.001). (B) Overall survival according to baseline NER. Higher NER (≥48) was significantly associated with shorter median OS (6.0 vs. 24.0 months, p = 0.002). NER - neutrophil-to-eosinophil ratio; PFS - progression-free survival; OS - overall survival.

During the follow-up period, 27 (55.1%) and 26 (53.1%) patients had disease progression and died from any cause, respectively. PFS and OS were compared according to baseline NER. Median PFS was significantly shorter in patients with high NER (NER ≥ 48) than in those with low NER (<48) (30.0 months (95% CI not available)) vs. (3.0 months (95% CI: 1.75-4.25), p < 0.001) (Figure 2A). In the low-risk group for OS (NER < 48), mOS was 24.0 months (95% CI: 4.5-43.5 months) and in the high-risk group (NER≥48), it was six months (95% CI: 3.8-8.2 months, p = 0.002). Therefore, mOS was also significantly shorter in patients with high NER (Figure 2B).

Univariable analysis showed that histopathology, NER, and occurrence of immune-related adverse events (irAEs) were significant factors for PFS (Table 3). Results of the multivariable analysis showed that only NER remained in the final model (HR 3.92 (95% CI: 1.66-9.23), p = 0.002).

**Table 3:** Univariable analyses for progression-free survival. PS ECOG - Eastern Cooperative Oncology Group Performance Status Scale; CI - confidence interval; HR - hazard ratio estimate; IMDC - International Metastatic Renal Cell Carcinoma Database Consortium; NER - neutrophil-to-eosinophil ratio; irAE - immune-related adverse events.

Variable	Univariable analysis HR (95% CI)	P-value
Age (years)		
≥65 vs. <65	0.98 (0.96-1.01)	0.307
Sex		
Male vs. female	0.95 (0.28-3.20)	0.937
PS ECOG		
0, 1 vs. 2	0.61 (0.27-1.40)	0.243
Previous nephrectomy		
Yes vs. no	0.49 (0.20-1.19)	0.113
Histopathology		
Non-clear cell vs. clear cell carcinoma	3.28 (1.36-7.89)	0.008
Fuhrman's grade		
1, 2 vs. 3, 4	1.45 (0.54-3.87)	0.457
IMDC risk		
Poor vs. favorable/intermediate	3.65 (0.86-15.58)	0.080
Number of previous therapies		
1 vs. ≥2	0.64 (0.19-2.14)	0.471
Number of metastatic organs		
≥2 vs. 1	1.09 (0.50-2.34)	0.833
NER		
≥48 vs. <48	3.92 (1.66-9.23)	0.002
Occurrence of irAE		
Yes vs. no	0.33 (0.12-0.91)	0.033
Delay nivolumab		
No vs. yes	0.78 (0.10-5.82)	0.807
Stop nivolumab		
No vs. yes	0.43 (0.10-1.85)	0.259

Univariable analysis showed that previous nephrectomy, irAE, NER, and progressive disease were significant factors for OS (Table 4). Multivariable analysis showed that NER (HR 3.85 (95% CI: 1.33-11.17), p = 0.013) and progressive disease (HR 5.62 (95% CI: 1.88-16.83), p = 0.002) were independent factors for OS. Those patients with NER ≥ 48 had approximately a four-fold higher risk of dying than those with NER < 48 (p = 0.013). Those patients whose disease progressed had approximately a six-fold higher risk of dying than those without disease progression (p = 0.002).

**Table 4:** Univariable and multivariable analyses for overall survival. PS ECOG - Eastern Cooperative Oncology Group Performance Status Scale; CI - confidence interval; HR - hazard ratio estimate; IMDC - International Metastatic Renal Cell Carcinoma Database Consortium; NER - neutrophil-to-eosinophil ratio; irAE - immune-related adverse events.

Variable	Univariable analysis HR (95% CI)	P-value	Multivariable analysis HR (95% CI)	P-value
Age (years)				
≥65 vs. <65	1.01 (0.98-1.05)	0.555	-	-
Sex				
Male vs. female	1.18 (0.35-3.96)	0.787	-	-
PS ECOG				
0, 1 vs. 2	1.31 (0.59-2.89)	0.510	-	-
Previous nephrectomy				
Yes vs. no	0.36 (0.15-0.85)	0.019	-	-
Histopathology				
Non-clear cell vs. clear cell carcinoma	1.55 (0.49-4.87)	0.453	-	-
Fuhrman's grade				
1, 2 vs. 3, 4	1.07 (0.34-3.35)	0.904	-	-
IMDC risk				
Poor vs. favorable/intermediate	1.81 (0.40-8.24)	0.442	-	-
Number of previous therapies				
1 vs. ≥2	1.45 (0.57-3.68)	0.436	-	-
Number of metastatic organs				
≥2 vs. 1	1.64 (0.74-3.63)	0.225	-	-
NER				
≥48 vs. <48	4.02 (1.49-10.86)	0.006	3.85 (1.33-11.17)	0.013
Disease progression				
Yes vs. no	6.66 (2.26-19.64)	0.001	5.62 (1.88-16.83)	0.002
Occurrence of irAE				
Yes vs. no	0.26 (0.09-0.79)	0.018	-	-
Delay nivolumab				
No vs. yes	0.55 (0.07-4.55)	0.576	-	-
Stop nivolumab				
No vs. yes	0.39 (0.09-1.71)	0.215	-	-

Objective response rate according to baseline NER

An association between baseline NER and ORR was evaluated. Table 5 shows that NER was significantly associated with ORR (NER ≥ 48 (12.5%) vs. NER < 48 (87.5%), p = 0.003).

**Table 5:** Objective response rate according to baseline NER. NER - neutrophil-to-eosinophil ratio; CR - complete response; PR - partial response; ORR - objective response rate.

Variable	CR	PR	ORR	P-value
NER				0.003
≥48	0 (0.0%)	2 (13.3%)	2 (12.5%)	
<48	1 (100.0%)	13 (86.7%)	14 (87.5%)	

Immune-related adverse events according to baseline NER

**Figure 3:**
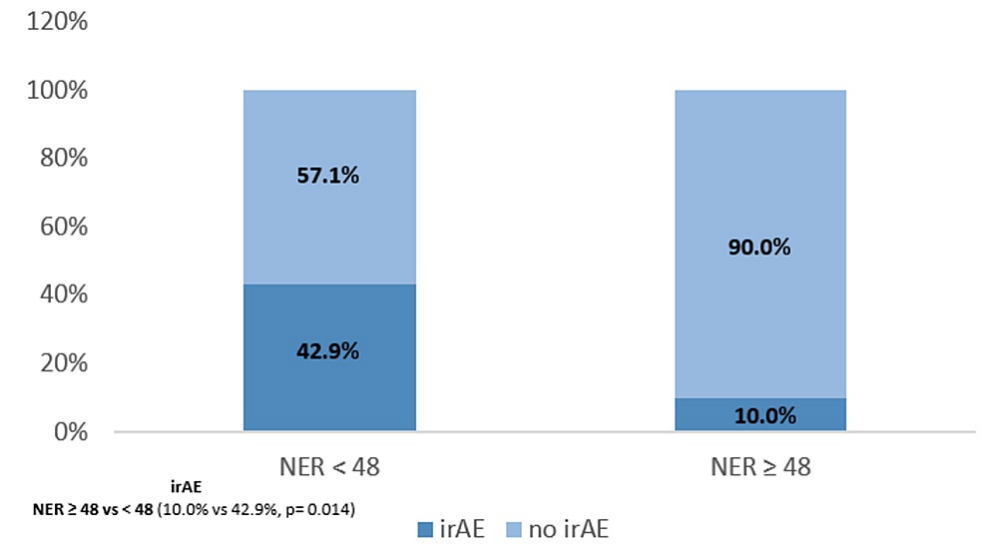
Immune-related adverse events according to baseline NER. NER - neutrophil-to-eosinophil ratio; irAE - immune-related adverse events.

An association between irAE and NER baseline was evaluated, which revealed that NER was significantly associated with irAE, and patients with NER ≥ 48 had fewer irAE than patients with NER < 48 (10.0% vs. 42.9%, p = 0.014) (Figure 3).

Histopathology according to baseline NER

**Figure 4:**
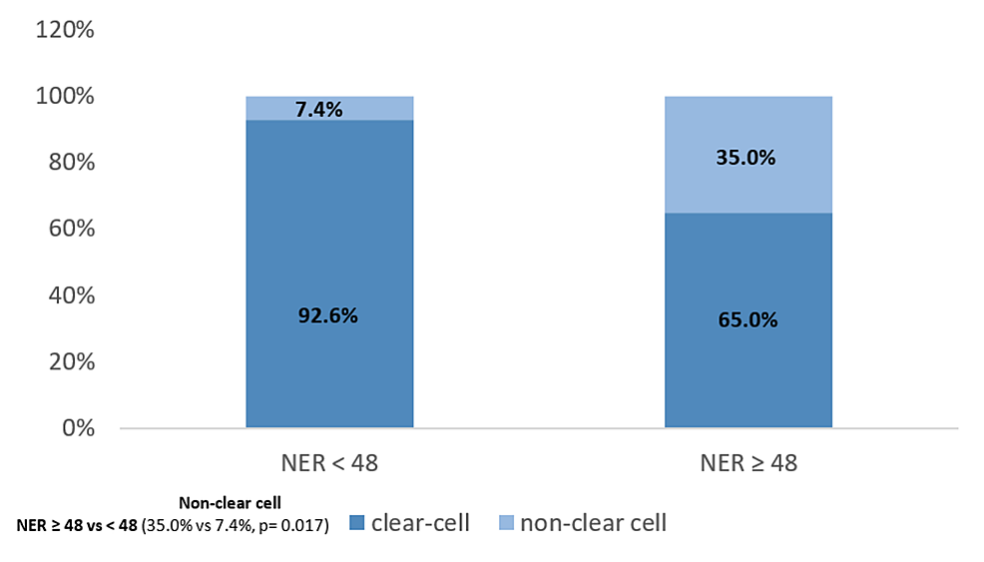
Histopathology according to NER baseline. NER - neutrophil-to-eosinophil ratio.

Association between histopathology and baseline NER was also evaluated, which revealed that NER was significantly associated with tumor histology and patients with NER ≥ 48 had more non-clear cell carcinoma than patients with NER < 48 (35.0% vs. 7.4%, p = 0.017) (Figure 4).

## Discussion

In recent decades, evidence has been presented establishing the association between inflammation and cancer development and several studies have suggested that inflammatory factors may be useful to predict survival in mRCC [5,13,19]. In this context, many inflammatory factors were investigated including the baseline NER associated with the results of immuno-oncology-based combination treatment in mRCC. A post hoc analysis of a phase III randomized control study, JAVELIN Renal 101, demonstrated that a lower NER was associated with a better ORR and PFS with the combination of avelumab and axitinib [21]. In addition to the association of NER and immuno-oncology-based combination treatment, this study also evaluated the possible value of this biomarker in patients treated with TKI monotherapy as the first line, revealing no significant difference for PFS in this subset of patients, but improvement in OS with lower NER. So, similarly to what has been documented in the NLR studies [13,24,25], NER may have a prognostic value in mRCC, regardless of the treatment type. Nevertheless, it seems to have an additional predictive value for patients treated with immunotherapy [21]. More recently, this was supported by the retrospective study in which Tucker et al. concluded that lower baseline NER was associated with better clinical outcomes (PFS, OS, and ORR) in patients with mRCC treated with nivolumab plus ipilimumab in the first line [26].

Therefore, our current retrospective analysis performed in patients with mRCC treated with nivolumab monotherapy in the second or later line represents an assessment of baseline NER as a possible prognostic biomarker. Our study provides real-world evidence for the impact of baseline NER when estimated prior to treatment with nivolumab in second or subsequent lines and demonstrated that a lower baseline NER was associated with an improvement in OS and PFS in these patients. This study also suggests an association between lower NER and better ORR. This is in agreement with previous studies [21,26]. Patients with a baseline NER ≥ 48 were considered high-risk groups, revealing a lower PFS and OS than the low group with a baseline NER < 48.

Two studies associated NER with survival and ORR outcomes, placing patients into low- and high-risk groups, using as cut-off values higher or lower than the median NER calculated for the sample around 26-29 [21,26], different from the cut-off obtained in our study. It applied an additive Cox proportional hazards model to identify the cut-off point for NER considering PFS. In multivariable Cox regression, baseline NER was considered an independent factor for PFS and OS in patients with mRCC treated with nivolumab in second or later lines. These results obtained from real-world data suggest that NER may be a potential prognostic factor for survival in patients with mRCC treated with nivolumab and support the hypothesis that this biomarker could be predictive of response to immunotherapy in patients with mRCC and of clinical utility. Looking at the literature, the hazard ratio associated with NER in our analysis is higher than in other published results [21,26]. Possible explanations for this discrepancy are the different statistical methods used to determine the NER cut-off and the fact that we have analyzed baseline NER pre-starting second or later lines with nivolumab. Our retrospective analysis also revealed a significant association between immune-related adverse events and NER baseline, as it showed that patients with higher NER tend to have less irAE than patients with lower NER. One retrospective study by Giorgione et al. published in February 2021 revealed that patients with mRCC who developed irAE had higher baseline eosinophil counts compared to those who did not, and this affects the degree of toxicity, which is higher for patients with higher baseline absolute eosinophils count [27]. Another retrospective study, which involved metastatic melanoma, mRCC, and metastatic non-small cell lung cancer receiving ICIs, showed that baseline eosinophil count may predict irAE and patients with baseline eosinophilia had more irAE [28]. This is related to the regulatory and effector role of eosinophils in multiple immune functions [27,28].

Another important finding from our retrospective analysis was the greatest tendency of patients with non-clear cell carcinoma to have higher NER. Considering the small number of patients with non-clear cell carcinoma histology, we cannot draw major conclusions from this result, so we believe it is pertinent to develop a study with a bigger sample and conduct a subgroup analysis of the different histology according to the NER and evaluating clinical outcomes.

This study has several limitations, including the fact that it is a retrospective study with a relatively short period of time of patient’s inclusion and that both neutrophils and eosinophils can be affected by a variety of situations including infection and medications, which makes the study even more limited to adequately explain the possible influences of these external factors. Another noteworthy limitation of this retrospective analysis is the fact that we did not perform a multivariable analysis to determine the predictive value of NER for the outcome ORR. We believe that the exploration of irAEs stratified by NER baseline in a larger sample and with subgroup analyses for the different types and degrees of toxicities is a hypothesis that should be researched in the future. This could add value to the role of the baseline NER as a predictor of irAE and understand the eventual association with better outcomes in the group of patients with lower baseline NER and who had more irAEs. Furthermore, the characterization of metastasis load, metastasis sites, and types of metastasis was not considered in our study. We recognize that the lack of this information may have led to biased results and should be considered in subsequent studies. Additionally, only about 18% of the patients in this study had non-clear cell histology, so we were limited in how the analysis of histology subgroups for PFS, OS, and ORR stratified by NER could result in an overestimation of the effect in such a small group. Combinations with immunotherapy are now preferred in the first line, therefore, we anticipate more real-world studies to confirm the predictive role of NER of important oncological outcomes also in the first line.

## Conclusions

The present real-world analysis using a multi-institutional cohort with mRCC treated with nivolumab in second or later lines confirmed the prognostic value of this biomarker, supporting its clinical utility in predicting survival. Results also suggested an association between lower NER and better ORR, and that immune-related adverse events occur more frequently in patients with a lower NER. Because this biomarker can be easily assessed and monitored in routine clinical practice, its use can contribute to effective treatment and follow-up of patients. However, further large-scale prospective studies are needed to confirm these findings and to validate this biomarker.

## Figures and Tables

**Figure 5:**
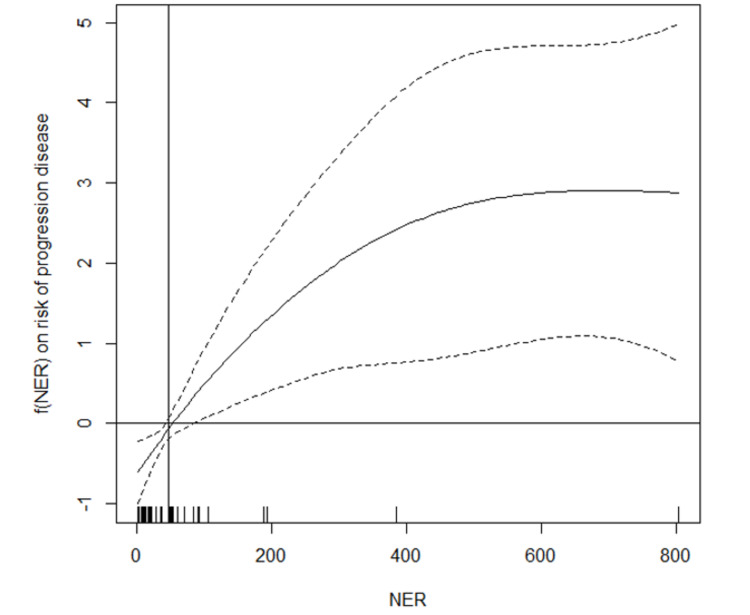
Supplemental Figure - Fitted smooth effects of NER on the risk of progression disease, represented by f(NER) with a black curve, with corresponding 95% confidence intervals (dashed lines) obtained by the additive Cox proportional hazard model. At a certain observed point of NER, a negative value of f(NER) means that, at that point, the expected risk of progression disease decreases. On the contrary, if f(NER) is positive, the expected risk of progression disease increases. The vertical black line identifies the NER cut-off of approximately 48. NER - neutrophil-to-eosinophil ratio.
